# Concurrent capecitabine and upper abdominal radiation therapy is well tolerated

**DOI:** 10.1186/1748-717X-1-41

**Published:** 2006-10-24

**Authors:** Prajnan Das, Robert A Wolff, James L Abbruzzese, Gauri R Varadhachary, Douglas B Evans, Jean Nicolas Vauthey, Andrew Baschnagel, Marc E Delclos, Sunil Krishnan, Nora A Janjan, Christopher H Crane

**Affiliations:** 1 Department of Radiation Oncology, The University of Texas M. D. Anderson Cancer Center, Houston, USA; 2 Department of Gastrointestinal Medical Oncology, The University of Texas M. D. Anderson Cancer Center, Houston, USA; 3 Department of Surgical Oncology, The University of Texas M. D. Anderson Cancer Center, Houston, USA

## Abstract

We retrospectively evaluated acute toxicity in 88 patients that were treated with capecitabine and concurrent radiotherapy to the upper abdomen. These patients included 28 (32%) with pancreatic adenocarcinoma, 18 (20%) with cholangiocarcinoma, 11 (13%) with ampullary carcinoma, 11 (13%) with other primary tumors, 14 (16%) with liver metastases, and 6 (7%) with metastases at other sites. The median dose of radiotherapy was 45 Gy (range 30–72 Gy). The median dose of capecitabine was 850 mg/m^2 ^twice daily, with 77% receiving 800–900 mg/m^2 ^twice daily. The highest grade of acute toxicity was Common Terminology Criteria (CTC) grade 0 in 5 (6%), grade 1 in 60 (68%), grade 2 in 18 (20%), and grade 3 in 5 (6%) patients. No patient had CTC grade 4 toxicity. The most common grade 2 toxicities were nausea, hand-foot syndrome, fatigue, anorexia and diarrhea. The grade 3 toxicities included nausea, vomiting and fatigue. Three patients (3%) required hospitalization due to grade 3 acute toxicity. Capecitabine was interrupted, discontinued or given at an adjusted dose in 13 (15%) patients because of acute toxicity. Therefore, capecitabine and concurrent radiotherapy to the upper abdomen appears to be well tolerated. Capecitabine may serve as an alternative to bolus or infusional 5-FU during chemoradiation for upper gastrointestinal malignancies.

## Findings

Capecitabine is an orally administered fluoropyrimidine that is preferentially converted to 5-FU in tumor tissue through a three-step enzymatic pathway[1]. Capecitabine is now widely used as an alternative to 5-FU for the treatment of gastrointestinal cancers. Randomized trials have shown that capecitabine gives at least equivalent outcomes as 5-FU and leucovorin for the treatment of metastatic colorectal cancer, as well as for adjuvant treatment of colon cancer [2-5]. Capecitabine may serve as an alternative to 5-FU for concurrent chemoradiation of gastrointestinal cancers. Phase I and II trials have shown that capecitabine is well tolerated with concurrent pelvic radiotherapy for rectal cancer, and yields pathologic complete response rates of 10–24% [6-10]. Small retrospective and prospective studies have previously reported that capecitabine is tolerated well with abdominal radiotherapy [11-14].

We retrospectively evaluated acute toxicity in 88 patients treated with concurrent capecitabine and radiation therapy to the upper abdomen, at the University of Texas M.D. Anderson Cancer Center, between June 2000 and July 2003. Patients who received a second concurrent chemotherapeutic agent along with capecitabine were excluded. Patients who received concurrent bevacizumab in addition to capecitabine and radiotherapy on a phase I protocol were also excluded from this study, and have been reported elsewhere[15]. The current study represents the largest reported series of patients treated with concurrent capecitabine and upper abdominal radiation therapy.

Chemoradiation was given as pre-operative treatment in 19 (22%), post-operative treatment in 24 (27%), definitive treatment in 5 (6%), and palliation in 40 (46%) patients. The median dose of radiation therapy was 45 Gy (range 30–72 Gy). Radiation therapy was given with 1.8–2 Gy fractions in 42 (48%) patients, 2.5 Gy fractions in 15 (17%) patients, and 3 Gy fractions in 31 (35%) patients. Radiation therapy was delivered by 6–18 MV photons with customized blocking. A two-field technique was used for 12 (14%), a three-field technique for 10 (11%), a four-field technique for 64 (73%), and intensity modulated radiation therapy for 2 (2%) patients.

Capecitabine was administered orally in twice-daily doses. The median dose of capecitabine was 850 mg/m^2 ^(range 400–900 mg/m^2^) twice daily. Sixty-eight (77%) patients received capecitabine at 800–900 mg/m^2 ^twice daily. There was clear documentation that capecitabine was given 5 days a week (Monday-Friday) in 47 (53%) patients, 6 days a week in 2 (2%) and 7 days a week in 3 (3%) patients. The frequency of capecitabine administration could not be reliably ascertained for the remaining 36 (41%) patients.

The median age of patients was 65.5 years (range 36.5–85.4 years). Of the 88 patients, 28 (32%) were treated for pancreatic carcinoma, 11 (13%) for ampullary carcinoma, 11 (13%) for extrahepatic cholangiocarcinoma, 8 (9%) for gall bladder cancer, 7 (8%) for intrahepatic cholangiocarcinoma, 3 (3%) for other primary tumors, 14 (16%) for liver metastases, and 6 (7%) for metastases at other sites.

Acute toxicity was graded using the Common Terminology Criteria for Adverse Events version 3.0. The highest grades of Common Terminology Criteria (CTC) acute toxicity during chemoradiation are shown in Table 1. The most common grade 2 toxicities were nausea, hand-foot syndrome, fatigue, anorexia and diarrhea. The grade 3 toxicities included nausea, vomiting and fatigue. No patient had any grade 4 toxicity. The highest grade of any acute toxicity during chemoradiation was grade 0 in 5(6%), grade 1 in 60 (68%), grade 2 in 18 (20%), and grade 3 in 5 (6%) patients.

**Table 1:** Highest Grades of Acute Toxicity During Chemoradiation

**Toxicity**	**Number of Patients (%)**		
	
	**Grade 1**	**Grade 2**	**Grade 3**
Nausea	50 (57)	9 (10)	3 (3)
Vomiting	18 (20)	1 (1)	4 (5)
Diarrhea	22 (25)	3 (3)	0 (0)
Hand-Foot Syndrome	2 (2)	4 (5)	0 (0)
Fatigue	39 (44)	4 (5)	2 (2)
Anorexia	24 (27)	3 (3)	0 (0)
Weight Loss	12 (14)	1 (1)	0 (0)
Constipation	12 (14)	1 (1)	0 (0)
Pain	24 (27)	1 (1)	0 (0)
Mucositis	5 (6)	0 (0)	0 (0)
Dehydration	2 (2)	2 (2)	0 (0)
Dysphagia	5 (6)	0 (0)	0 (0)
Heartburn	2 (2)	0 (0)	0 (0)
Skin	7 (8)	0 (0)	0 (0)
Anemia	6 (7)	2 (2)	0 (0)
Leukopenia	1 (1)	0 (0)	0 (0)
Thrombocytopenia	3 (3)	0 (0)	0 (0)
Other	3 (3)	2 (2)	0 (0)

Five patients required hospitalization during or immediately after chemoradiation, of whom 3 (3%) were hospitalized due to acute toxicity. A radiation treatment break of 1 day was required in 3 patients, and radiotherapy was stopped early in 1 patient. Capecitabine administration was modified in 13 (15%) patients because of acute toxicity. These modifications included discontinuation of capecitabine (n = 2), a break in capecitabine (n = 4), a break followed by dose reduction of capecitabine (n = 4), and dose reduction without a break (n = 3).

Our results, therefore, indicate that upper abdominal radiation therapy was well tolerated with concurrent capecitabine. Capecitabine has potential advantages over bolus or protracted infusional 5-FU for concurrent chemoradiation. Since capecitabine is orally administered, its advantages include convenience and ease of administration. Studies have demonstrated that patients prefer oral chemotherapy to intravenous chemotherapy as long as efficacies are comparable[16,17]. Capecitabine has been shown to decrease the use of medical resources, compared to bolus 5-FU[18]. Moreover, capecitabine obviates the need for a venous catheter, which could be associated with a risk for venous thrombosis and line infections. However, since capecitabine is self-administered, its efficacy depends on patient compliance. Moreover, capecitabine is contraindicated in certain groups of patients such as those with severe renal dysfunction. Capecitabine also produces interactions with certain drugs such as coumadin and phenytoin.

Patients treated with capecitabine and concurrent chemoradiation should be monitored closely for acute toxicity. Patients who start developing acute toxicity often need adjustments in capecitabine, such as dose reduction, treatment break or discontinuation of capecitabine. As many as 15% of patients in this study underwent modifications in capecitabine during chemoradiation. Careful monitoring of patients likely played an important role in limiting the rates of acute toxicity in this study. At our institution, monitoring of these patients includes weekly blood counts and weekly assessment of diarrhea and hand-foot syndrome.

Our results are comparable to previous, smaller studies on radiation therapy with concurrent capecitabine. Vaishampayan et al. reported a retrospective study on 32 patients treated with capecitabine and radiotherapy to various sites, including abdominal radiotherapy[11]. Grade 3–4 toxicities included neutropenia in 3 patients, and diarrhea, thrombocytopenia, fatigue and myocardial infarction, each in 1 patient. Ben-Josef et al. reported a retrospective study on 15 patients with pancreatic cancer treated with concurrent capecitabine and intensity modulated radiotherapy[12]. Eight patients (53%) had grade 1–2 nausea/vomiting, and only 1 patient had grade 3 toxicity. Saif et al. performed a phase I study of radiation therapy with concurrent capecitabine in 15 patients with pancreatic cancer[14]. No dose limiting toxicities were seen at capecitabine dose levels of 600 and 800 mg/m^2 ^twice daily, but 2 of 6 patients experienced grade 3 diarrhea at a dose level of 1000 mg/m^2 ^twice daily. Schneider et al. performed a prospective study of capecitabine and radiotherapy, preceded and followed by chemotherapy, in patients with pancreatic cancer[13]. Nineteen patients received chemoradiation in this study, of whom 1 had grade 3 nausea/vomiting, 1 had grade 3 diarrhea, 1 had grade 3 fatigue, 2 had grade 3 infectious colitis, and 1 had grade 3 rash. These studies together indicate that capecitabine is well tolerated with abdominal radiation therapy.

The current study has certain limitations. Acute toxicity was assessed retrospectively based on a review of medical records. Hence, the rates of acute toxicity may have been under-estimated. The patient population was heterogeneous with a range of tumor sites. Patients were treated with a range of radiotherapy doses and capecitabine doses. However, 77% of patients received capecitabine at 800–900 mg/m^2 ^twice daily, and the majority of patients received capecitabine 5 days a week, only on the days of radiotherapy.

In conclusion, this large single-institution retrospective study indicates that upper abdominal radiation therapy was well tolerated with concurrent capecitabine at a dose of 800–900 mg/m^2 ^twice daily on days of radiation treatment. Only 6% of patients had grade 3 acute toxicity and no patient had grade 4 acute toxicity during chemoradiation. Moreover, only 3% of patients required hospitalization due to acute toxicity. Capecitabine may, therefore, serve as an alternative to bolus or infusional 5-FU during chemoradiation for upper gastrointestinal malignancies. Patients need to be monitored closely during chemoradiation with capecitabine, since some patients require adjustments in capecitabine dosing during chemoradiation.

## Competing interests disclosure

RAW has served on the Speakers' bureau for Roche, NAJ has received research funding from Roche, and CHC has received honoraria from Roche.

## Authors' contributions

PD and CHC conceived of the study, coordinated the study and helped to draft the manuscript. AB participated in data analysis. RAW, JLA, GRV, DBE, JNV, AB, MED, SK, and NAJ participated in data collection. All authors read and approved the final manuscript.
